# Semi-Coprime Array with Staggered Beam-Steering of Sub-Arrays

**DOI:** 10.3390/s23125484

**Published:** 2023-06-10

**Authors:** Waseem Khan, Saleem Shahid, Waleed Iqbal, Ahsan Sarwar Rana, Hijab Zahra, Moath Alathbah, Syed Muzahir Abbas

**Affiliations:** 1 Department of Electrical and Computer Engineering, Air University, Islamabad 44000, Pakistan; 2 Department of Avionics Engineering, Air University, Islamabad 44000, Pakistan; 3 School of Engineering, Faculty of Science and Engineering, Macquarie University, Sydney, NSW 2109, Australia; 4 College of Engineering, King Saud University, Riyadh 11451, Saudi Arabia; 5 School of Engineering, Cardiff University, Cardiff CF24 3AA, UK

**Keywords:** beam-pattern, linear antenna array, semi-coprime array, split-aperture array, staggered steering

## Abstract

A split-aperture array (SAA) is an array of sensors or antenna elements in which the array is split into two or more sub-arrays (SAs). Recently proposed SAAs, namely coprime and semi-coprime arrays, offer to attain a small half-power beamwidth (HPBW) with a small number of elements, compared to most conventional unified-aperture arrays, at the cost of reduced peak-to-side-lobe ratio (PSLR). To reduce HPBW and increase PSLR, non-uniform inter-element spacing and excitation amplitudes have proven helpful. However, all the existing arrays and beam-formers suffer increased HPBW, degraded PSLR or both when the main beam is steered away from the broadside. In this paper, we propose staggered beam-steering of SAs, a novel technique for decreasing HPBW. In this technique, we steer the main beams of the SAs of a semi-coprime array to angles slightly different from the desired steering angle. In conjunction with staggered beam-steering of SAs, we have utilized Chebyshev weights to suppress the side lobes. The results show that the beam-widening effect of Chebyshev weights can be mitigated considerably by staggered beam-steering of the SAs. Ultimately, the unified beam-pattern of the whole array offers HPBW and PSLR better than the existing SAAs, uniform and non-uniform linear arrays, especially when the desired steering angle is away from the broadside direction.

## 1. Introduction

The design of an array of antennas or sensors has been the area of research for a long time with the objective of achieving a half-power beam-width (HPBW) as small as possible, peak-to-side lobe ratio (PSLR) as high as possible, and using as few antenna elements as possible. To achieve this objective, researchers have analysed different geometries, inter-element distances, aperture sizes and excitation amplitudes or weights. Linear geometry has been tested for uniform [1,2,3,4] and non-uniform distances [5,6,7,8,9,10,11,12,13,14]. Uniform linear arrays (ULAs) have been investigated for different inter-element spacing ranging from a fraction to few multiples of a wavelength (λ). The most common ULA, called a standard ULA (SULA) hereafter, has a standard spacing of λ/2. Larger spacing causes larger aperture offering smaller HPBW, i.e., better resolution but spacing more than λ/2 produces grating lobes which are not desirable. On the other hand, increasing the aperture size while maintaining the standard spacing requires more elements which means higher system costs. To increase the aperture size with fewer elements (*L*), while avoiding grating lobes, non-uniform linear arrays (NULAs) were proposed. One of the techniques to create an NULA is sparsification or thinning of the SULA in which many of the elements are removed. Other methods for designing NULAs include finding inter-element spacing using either a non-linear function such as a logarithm [5], parabolic relation [13], etc., or a heuristic optimization technique such as genetic algorithm (GA) [8], firefly algorithm (FA) [6], particle swarm optimization (PSO) [15], invasive weed optimization (IWO) [9], chicken swarm optimization (CSO) [16], grasshopper optimization (GO) [17], etc. To achieve either a small HPBW or high PSLR, a number of non-uniform weighting techniques have been applied to both ULAs and NULAs. These analytical techniques include Dolph–Chebyshev [4], binomial, Blackman [1,18], Hanning [5], etc., as well as heuristic optimization algorithms such as PSO [10,11], IWO [3], strawberry algorithm [12]. The Dolph–Chebyshev technique pioneered by C.L. Dolph [19] in the first half of the 20th century is still employed in advanced antenna systems. For instance, in adaptive beam-forming, a method of calculating initial weights was proposed using Chebyshev distribution which suppressed the side lobes in the 0–30 dB range [20]. A modified Chebyshev window was also developed for a non-linear frequency-modulated waveform [21]. This modified Chebyshev window was able to suppress the peak side lobe level ratio by approximately −41 dB and integrated side lobe level ratio by approximately −31 dB. The Dolph–Chebyshev slow-wave transmission line feed networks have also been implemented, achieving side lobe levels of −30 dB [22]. In other research, iterative Fourier transform and Chebyshev-based random thinning were presented to lower the side lobe. However, this is preferred for small arrays only where it can suppress the side lobe level by −36.9 dB at 29° [23]. Apart from weighting techniques, there are various alternative approaches reported in the literature that leverage advanced antenna designs and meta-surface technologies to enhance the PSLR. For instance, circularly polarized antenna arrays in [24] refer to an antenna configuration that utilizes not only circularly polarized elements but also decoupled quad vortex beams to steer the main beam and control the side lobe levels. Chirality-assisted high-efficiency meta-surfaces utilize the concept of manipulating the chirality in electromagnetic waves to design efficient and beam-steerable antennas [25]. These meta-surfaces employ sub-wavelength structures created to control the radiation properties, including the suppression of side lobes. Dual-mode transmissive meta-surfaces operate in two distinct modes, usually with orthogonal polarizations or different frequencies [26]. The different scattering response for each mode is utilized to shape the radiation pattern and suppress the side lobes.

A linear array, in any case, conventionally has been treated as a single array having single beam pattern. However, a decade ago a coprime sensors array (CSA) was proposed [27], in which an NULA was split into two sub-arrays (SAs). In this SAA, the two SAs with large inter-element spacing were used to generate two different beam patterns. To unify the two beam patterns, in the pioneering work [27], their product was taken, but in subsequent works, their minimum [28] and hybrid [29,30] were also been considered. The CSA offers a small HPBW with fewer elements compared to SULA but a PSLR poorer than SULA. To enhance the PSLR, in [18,31,32], more sensors were added to the CSA, thus forming an extended CSA (ECSA). Recently, a modified version of the CSA, called the semi-coprime array (SCA) was proposed [33] in which an NULA is split into three SAs. Without compromising the HPBW, this array offers a PSLR higher than the CSA but still lower than SULA.

It should be noted that all the arrays discussed above offer their best performance metrics, i.e., HPBW and PSLR, when steered in the broadside direction. As the main beam is steered away from the broadside, the performance of all the arrays worsens, either in terms of HPBW, PSLR, or both. In some cases directivity also decreases when the main beam is steered away from the broadside. A few existing arrays offer the same HPBW/PSLR for different look angles but require the elements to be relocated when steered to a new angle [11,34]. However, in most scenarios, it is not possible to relocate them at the run time.

In this paper, we propose staggered beam-steering of SAs in an SAA. Simulation results show that beam-steering the SAs slightly away from the desired steering angle can help generate a narrower beam pointed towards the desired angle, if the minimum of the beam patterns is taken to unify them. In combination with this technique, Chebyshev weights have been employed to improve the PSLR. For the proof of concept, we have applied staggered steering on an SCA only with a minimum processor. However, it can be applied to any SAA. We have shown in this paper, that an SCA with staggered steering (SCASS) outperforms the existing ULAs, NULAs and SAAs in terms of HPBW, PSLR and directivity, especially when steered away from the broadside. The rest of the paper is organized as follows. Section 2 describes the basic and proposed forms of an SCA. Section 3 presents the simulation results of the proposed array and compares it with different existing arrays. Finally, Section 4 discusses the simulation results and concludes the paper.

## 2. SCA: Basic and Proposed Forms

In this section, we first present the original or basic form of an SCA and then we describe the proposed variant of the SCA.

### 2.1. SCA

**Figure 1:**
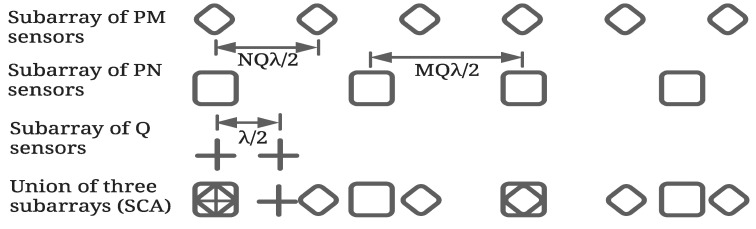
A typical arrangement of an SCA with *M* = 3, *N* = 2, *P* = 2 and *Q* = 2.

SCA, proposed in [33], is the union of three SAs called hereafter SA1, SA2 and SA3 having PM, PN and *Q* elements with spacing QNλ/2, QMλ/2 and λ/2, respectively. Figure 1 depicts a typical set of SAs of an SCA. Array factor (AF) of the *i*th SA of an SCA can be expressed as:

(1) $$AF_{i}\left(\theta \right)=\sum_{n}^{}w_{n,i}e^{j\left(n-1\right)kd_{i}\sin\theta },i=1,2,3$$

where θ is the angle with respect to the broadside direction of the array, $d_{1}=QN\lambda$/2, $d_{2}=QM\lambda$/2 and $d_{3}=\lambda$/2, and $w_{n,i}$ is the weight of the *n*th element of the *i*th SA. Here, *P*, *Q*, *M* and *N* are positive integers and *M* and *N* are coprime. It may be noted that the total number of elements is *L*=PM+PN+Q−1−P.

In the basic SCA, the three SAs are employed with uniform excitation amplitudes or weights, to generate beam patterns, steered towards angle $\theta_{0}$. It should be noted that all the SAs are beam-steered towards the same angle $\theta_{0}$. To unify the three patterns, in [33], a minimum processor was employed; therefore, the overall beam pattern of an SCA can be expressed as:

(2) $$B\left(\theta \right)=\min\left(|AF_{1}{\left(\theta \right)|}^{2},|AF_{2}{\left(\theta \right)|}^{2},{\left|AF_{3}\left(\theta \right)\right|}^{2}\right)$$

### 2.2. SCA with Staggered Steering (SCASS)

In the proposed variant of the SCA, the basic SCA structure, as described above, is employed with staggered beam-steering of SA1 and SA2. To be specific, if the desired steering angle of the whole array is $\theta_{0}$, then the SAs 1, 2 and 3 are beam-steered towards $\theta_{1}=\theta_{0}+\Delta$, $\theta_{2}=\theta_{0}-\Delta$ and $\theta_{3}=\theta_{0}$, respectively. Here, Δ is the deviation of the steering angle from $\theta_{0}$ and is a configurable parameter. On the contrary, in CSA and SCA, all the SAs are beam-steered towards $\theta_{0}$. SCASS has been investigated here in two forms, namely (i) SCASS with uniform weights (SCASS-U) and (ii) SCASS with Chebyshev weights (SCASS-C). In the first form, uniform weights are employed in all the SAs; while in the second form, Chebyshev weights are employed in SAs 1 and 2 and uniform weights are employed in SA3. Array factors of the three SAs cannot be expressed in a closed-form for Chebyshev weights; however, for uniform weights the array factors are:

(3) $$AF_{1}\left(\theta \right)=\frac{1}{PM}\left[\frac{\sin\left(\frac{PMQN\pi }{2}\left\{\sin\theta -\sin\left(\theta_{0}+\Delta \right)\right\}\right)}{\sin(\frac{1}{2}QN\pi \left(\sin\theta -\sin\left(\theta_{0}+\Delta \right)\right))}\right]$$

(4) $$AF_{2}\left(\theta \right)=\frac{1}{PN}\left[\frac{\sin\left(\frac{PMQN\pi }{2}\left\{\sin\theta -\sin\left(\theta_{0}-\Delta \right)\right\}\right)}{\sin(\frac{1}{2}QM\pi \left(\sin\theta -\sin\left(\theta_{0}-\Delta \right)\right))}\right]$$

(5) $$AF_{3}\left(\theta \right)=\frac{1}{Q}\left[\frac{\sin(\frac{Q}{2}\pi \left(\sin\theta -\sin\theta_{0}\right))}{\sin(\frac{1}{2}\pi \left(\sin\theta -\sin\theta_{0}\right))}\right]$$

Both forms have been investigated for different steering angles. The value of Δ is selected for each steering angle $\theta_{0}$, such that HPBW and PSLR remain either the same or degrade minimally when the main beam is steered away from the broadside direction. This contrasts with the existing ULAs and NULAs in which the HPBW and PSLR degrade considerably when the beam is steered away from the broadside.

## 3. Simulations and Results

### 3.1. SCASS-U

**Figure 2:**
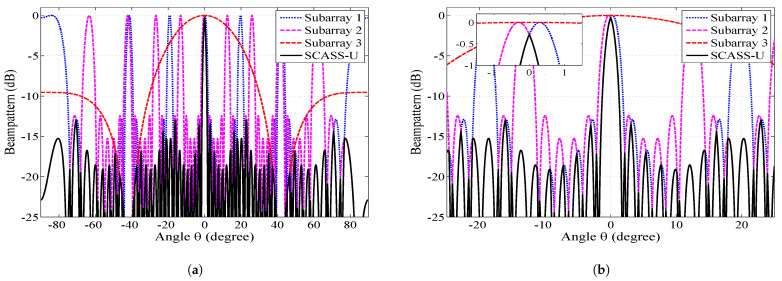
Beam pattern of the SCASS-U and its SAs with *M* = 3, *N* = 2, *P* = 3, *Q* = 3 (*L* = 14) and $\Delta =0.3^{\circ }$: (**a**) whole view (**b**) partial view.

SCASS-U was simulated with 14 elements, steered towards the broadside with $\Delta =0.3^{\circ }$. Figure 2 shows the beam patterns of the SAs and their unified beam pattern. It is clear in Figure 2b that the main lobe of the SCASS-U is narrower than the main lobes of the SAs. This is because the main lobe of the overall beam pattern is, in fact, the overlapping portion of the main lobes of SA1 and SA2. It can seen from the figure that an increase in Δ (note that the separation between the maxima of the beam pattern of SA1 and SA2 is 2Δ) will definitely reduce the overlapping region, thus consequently reducing the width and height of the main lobe of the overall beam pattern. Hence, for the selected Δ (0.3${}^{\circ }$), the main lobe of the SCASS-U is 0.28 dB lower than the main lobes of the SAa. This represents a power loss of 0.28 dB. It is obvious from the discussion above that an increase in Δ will cause a narrower main lobe but a higher power loss. Therefore, Δ needs to be kept small so that the power loss remains within a limit. This power loss also causes the PSLR to degrade. To regain the PSLR, by suppressing the side lobes, Dolph–Chebyshev weights have been employed to form an SCASS-C, discussed in the next subsection. It is also noteworthy here that in Figure 2, the grating lobes of SA1 and SA2 do not overlap. However, if Δ is large, the grating lobes overlap, causing higher side lobes and a lower PSLR. Thus, PSLR degradation with increasing Δ is due to two factors; power loss in the main lobe and overlapping of the grating lobes. The first factor causes the peak of the main lobe to decrease while the second causes the side lobes to rise. Power loss is always there for all non-zero values of Δ while the grating lobes only overlap for large values of Δ.

### 3.2. SCASS-C

SCASS-C with Chebyshev weights has been simulated for *L* = 14, steered towards different angles with suitably selected values of Δ. Employing Chebychev weights in linear arrays is a conventional technique to suppress side lobes of the beam pattern, consequently improving the PSLR. Although this benefit is accompanied with increased HPBW, in SCASS-C, this drawback is alleviated by adjusting the value of Δ. In this way, despite the power loss, PSLR is improved without compromising the HPBW. This is in contrast with existing linear arrays in which the HPBW is compromised when the PSLR is improved and vice versa. Chebyshev weights are calculated for a given side lobe attenuation (SLA) [35]; therefore, in the results shown here, the SLA values are also mentioned.

**Figure 3:**
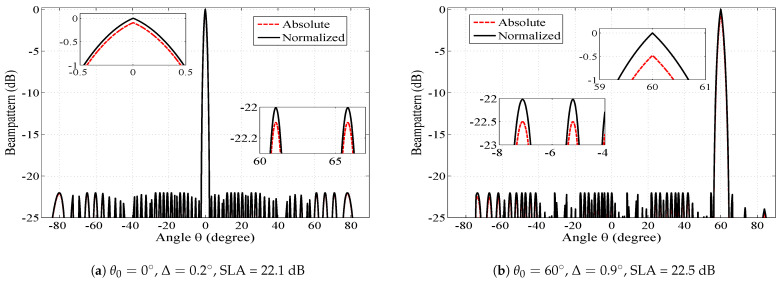
Absolute and normalized beam patterns of SCASS-C, *M* = 3, *N* = 2, *P* = 3, *Q* = 3 and power loss = (**a**) 0.1 dB, (**b**) 0.5 dB.

Figure 3 shows the absolute and normalized beam pattern of the SCASS-C with 14 elements. Here, the adjustable parameters are set as Δ = 0.2${}^{\circ }$, 0.9${}^{\circ }$ and SLA = 22.1 dB, 22.5 dB for $\theta_{0}$ = 0${}^{\circ }$, 60${}^{\circ }$, respectively. Note that the peak side lobe level of the absolute beam pattern is the same as the given SLA, but the beam pattern peak is lower than 0 dB. For instance, for $\theta_{0}$ = 60${}^{\circ }$, the peak side lobe level is −22.5 dB while the beam pattern peaks at −0.5 dB. Hence the PSLR becomes 22 dB. As shown in the figure, when the beam pattern is normalized, its peak and side lobes increase identically. Consequently, its PSLR remains the same in the absolute and normalized patterns. Therefore, in all the figures, from this point onward, only the normalized beam patterns are plotted for easy comparison with the beam patterns of various existing arrays.

#### Comparison of the SCASS-C with Existing Arrays

**Figure 4:**
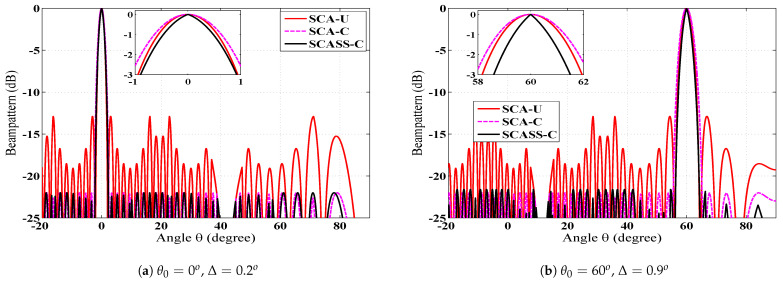
Beam pattern of the SCASS-C, SCA-U and SCA-C, *M* = 3, *N* = 2, *P* = 3 and *Q* = 3 (*L* = 14); for the SCA-C, SLA = 22 dB; for SCASS-C, (**a**) SLA = 22.1 dB, power loss = 0.1 dB, (**b**) SLA = 22.5 dB, power loss = 0.5 dB.

Since SCASS-C is the modified version of the SCA, it is first compared with the basic SCA in Figure 4. This figure shows the beam patterns of the SCASS-C, SCA with uniform weights (SCA-U) and SCA with Chebyshev weights (SCA-C). Although the basic SCA employs uniform weights, we have simulated an SCA with Chebyshev weights for a fair comparison. It is evident from the figure that the SCASS-C offers an HPBW smaller than SCA-U and SCA-C, while the PSLR of both the SCASS-C and SCA-C is 22 dB. Here, the power loss in the SCASS-C, for $\theta_{0}=0^{\circ }$ and $\theta_{0}=60^{\circ }$, is 0.1 and 0.5 dB, respectively. Note that for the SCA-C, SLA = 22 dB and for the SCASS-C, SLA = 22.1 dB, 22.5 dB for $\theta_{0}=0^{\circ }$, $60^{\circ }$, respectively. Therefore, despite the power loss, the SCASS-C gives a PSLR of 22 dB for the two steering angles. If the SLA is set to 22.1 dB or 22.5 dB for the SCA-C too, its HPBW degrades further which is already worse than both the SCA-U and SCASS-C. Hence, the SCASS-C outperforms the SCA-U and SCA-C in terms of the HPBW for a given PSLR.

**Figure 5:**
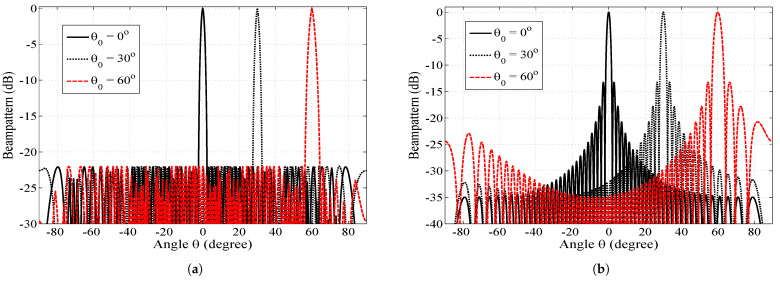

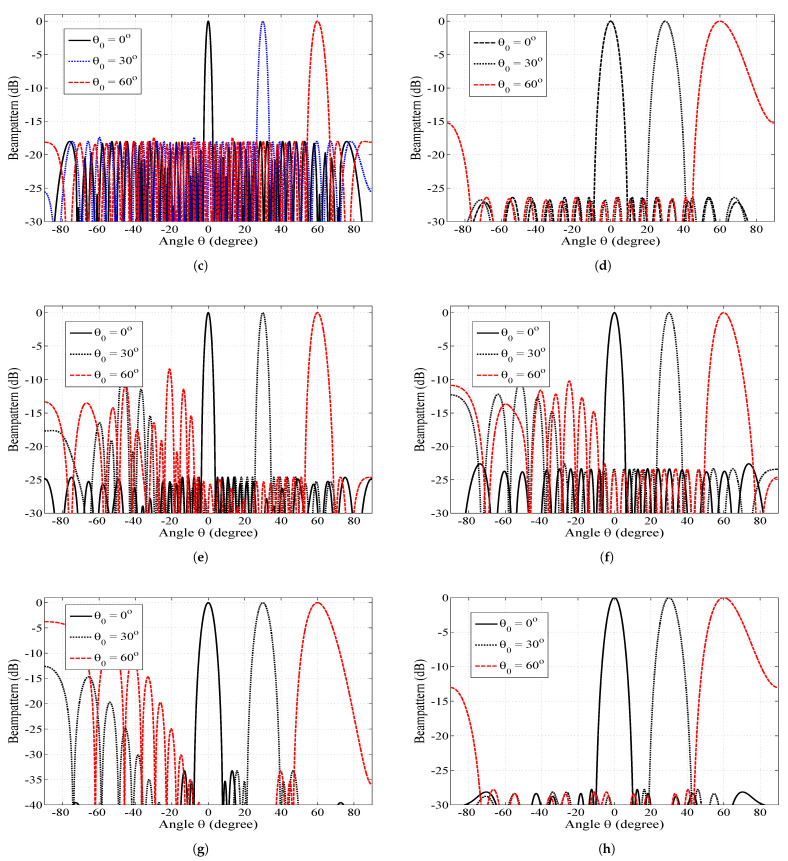
Normalized beam pattern of different ULAs and NULAs for $\theta_{0}=0^{\circ }$, $30^{\circ }$, $60^{\circ }$. (**a**) (M,N,P,Q) = (3,2,3,3), and SLA = 22.1, 22.15, 22.5 dB, $\Delta =0.2^{\circ },\;0.3^{\circ },\;0.9^{\circ }$ for $\theta_{0}=0^{\circ },\;30^{\circ },\;60^{\circ }$, respectively. (**b**) (M,N) = (7,8) (**c**) (M,N) = (3,4), extension factor = 3. (**a**) SCASS-C, *L* = 14. (**b**) CSA-N, *L* = 14 [30]. (**c**) ECSA-C, SLA = 18 dB, *L* = 18 [18]. (**d**) SULA-IWO, *L* = 16 [3]. (**e**) NULA-GO, *L* = 32 [17]. (**f**) NULA-GA, *L* = 20 [14]. (**g**) NULA-P, *L* = 21 [13]. (**h**) SULA-PSO, *L* = 16 [10].

In the second step, the proposed array is compared with other existing arrays. Figure 5 shows the beam pattern of the SCASS-C compared against different existing ULAs, NULAs and SAAs, namely: (i) CSA with a novel processor (CSA-N) [30]; (ii) ECSA with Chebyshev weights (ECSA-C) [18]; (iii) SULA with IWO-optimized weights (SULA-IWO) [3]; (iv) NULA with GO-optimized spacing (NULA-GO) [17]; (v) NULA with GA-optimized spacing (NULA-GA) [8]; (vi) NULA with spacing and weights found using parabolic relations (NULA-P) [13]; and (vii) SULA with PSO-optimized weights (SULA-PSO) [10]. For simulation of the existing arrays, their weights, spacings and other parameters were taken as proposed originally. However, for the ECSA-C, we took *M* = 3, *N* = 4 and the extension factor = 3. It is obvious from these figures that all the arrays suffer degradation either in the HPBW, PSLR or both when they are beam-steered away from the broadside. In contrast, the HPBW of SCASS-C degrades minimally among them while its PSLR remains constant when steered away from the broadside.

The performance metrics of these arrays are shown in Table 1. This table shows that the SCASS-C, with suitably selected Δ and SLA, offers the minimum HPBW among them, except for CSA-N [30] which offers an HPBW 0.02${}^{\circ }$ smaller than the SCASS-C in the broadside direction. However, as soon as it is beam-steered away from the broadside its main lobe becomes wider than the SCASS-C. Moreover, its PSLR is inferior to that of the SCASS-C for all steering angles. Although some arrays are superior to the SCASS-C in terms of the PSLR for $\theta_{0}=0^{\circ }$, when they are beam-steered away from the broadside, not only do their HPBW worsen further but their PSLR also drop below that of the SCASS-C. It is also noteworthy that the SCASS-C has been simulated for 14 sensors, while the other arrays have been simulated for a larger number of sensors, except CSA-N. Even then, the SCASS-C outperforms the other arrays in terms of its HPBW and directivity for the angles away from the broadside direction. The only array with a directivity higher than the SCASS-C is the CSA-N. However, at most, it is only 0.24 dB more directive than the SCASS-C; on the other hand, its PSLR is 8.75 dB lower than the SCASS-C. In Table 1, the performance metrics of NULA with PSO-optimized spacing and uniform weights [10] are also shown in the last row. This array has not been simulated in this research work and its HPBW and PSLR values have been taken from Table 3 in [10]. It is obvious that its PSLR and HPBW are inferior to the proposed SCASS-C even for the broadside direction. If steered away from the broadside, its performance worsens even further. Hence, only the proposed array can maintain all three performance metrics at decent values for all the given steering angles. A noteworthy fact is that the SCASS-C uses Δ as a tool to control the HPBW; the greater Δ, the smaller the HPBW. If this tool is used in conjunction with Chebyshev weights, HPBW and PSLR are both improved. This is in contrast to the existing arrays in which if one of the parameters is improved while the other suffers.

## 4. Parametric Analysis

It has been mentioned above that increasing Δ reduces the HPBW. But another effect of increasing Δ is its increase in power loss causing the PSLR to degrade. To regain the PSLR by suppressing the side lobes further, SLA of Chebyshev weights can be further increased. However, suppressing the side lobes means widening the SA main and grating lobes of the SCASS-C. Thus, if SLA is increased beyond a limit, the grating lobes of SA1 and SA2 overlap, considerable increasing the side lobes of the overall beam pattern. Therefore, Δ and SLA can be manipulated, up to certain limits, to improve the HPBW and PSLR. The effect of these parameters on the performance metrics has been analysed below.

### 4.1. Effect of Δ for Fixed *L* and SLA

**Figure 6:**
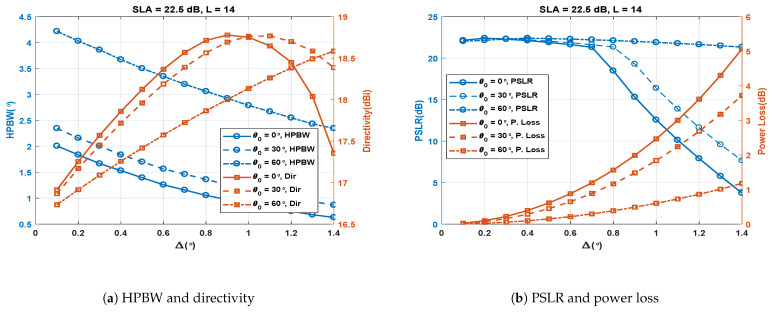
Performance metrics of the SCASS-C for variable Δ, fixed SLA = 22.5 dB and *L* = 14.

The simulations were carried out to study the effect of Δ with three different cases of $\theta_{0}$ (0${}^{\circ }$, 30${}^{\circ }$ and 60${}^{\circ }$). The SLA was fixed at 22.5 dB and the number of antenna elements (*L*) was fixed at 14. Figure 6a shows the results for Δ vs. HPBW and directivity. It is obvious from the figure that HPBW decreases almost linearly with an increase in Δ for all the values of $\theta_{0}$. HPBW also increases when $\theta_{0}$ is increased from 0${}^{\circ }$ to 60${}^{\circ }$. The figure also shows that directivity increases almost linearly with increasing Δ until Δ reaches a certain limit, after which directivity decreases rapidly. This limit is larger for larger values of $\theta_{0}$. Thus, for $\theta_{0}$ = 0${}^{\circ }$ and 30${}^{\circ }$, directivity is at a maximum at Δ = 0.9${}^{\circ }$ and 1.1${}^{\circ }$, respectively. For $\theta_{0}$ = 60${}^{\circ }$, the point of maximum directivity is even beyond the range of Δ shown in the figure. The initial increase in directivity with increasing Δ is due to the reducing beamwidth. When Δ is increased further, the beam patterns of SA1 and SA2 are staggered to an extent that their grating lobes start to overlap. This phenomenon causes side lobes to rise and the PSLR to degrade, consequently reducing directivity. This effect on the PSLR is shown in Figure 6b. It is evident from this figure that the PSLR remains almost constant as Δ increases, until it reaches a certain limit, after which the grating lobes overlap and the PSLR drops sharply. Figure 6b also shows that the power loss increases exponentially as Δ increases. Moreover, the rate of exponential growth also increases as $\theta_{0}$ increases from 0${}^{\circ }$ to 60${}^{\circ }$. The effect of Δ on the performance metrics can be understood keeping the fact in view that Δ controls the overlapping region of the main lobes of SA1 and SA2 if Δ is kept small, otherwise it also causes and controls the overlapping of their grating lobes. The larger Δ is, the narrower the overlapping region of the main lobes causing a smaller HPBW and a higher power loss.

### 4.2. Effect of SLA for Fixed Δ and *L*

**Figure 7:**
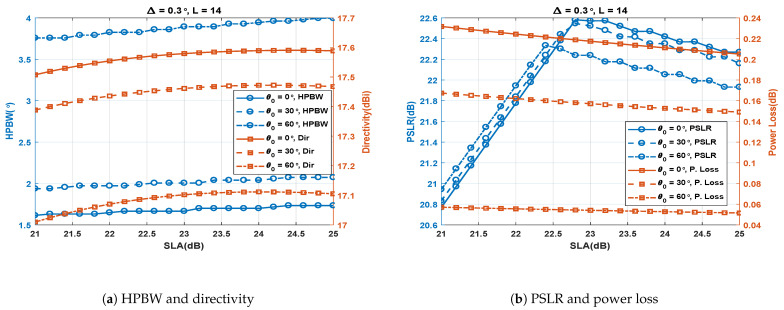
Performance metrics of the SCASS-C for variable SLA, fixed Δ =0.3${}^{\circ }$ and *L* = 14.

The simulations were carried out to study the effect of SLA with three different cases of $\theta_{0}$ (0${}^{\circ }$, 30${}^{\circ }$ and 60${}^{\circ }$). The Δ was fixed at 0.3${}^{\circ }$ and the number of antenna elements (*L*) is fixed at 14. Figure 7a shows the results for SLA vs. HPBW and directivity. The figure shows that with an increase in SLA, HPBW and directivity increase slightly. Increasing SLA causes widening of the SA1 and SA2 main lobes, thus increasing their overlapping region and consequently increasing the HPBW and reducing the power loss, i.e., increasing the PSLR. Increasing HPBW and PSLR have opposite effects on directivity; however, the PSLR has a slightly dominant effect, and therefore the directivity increases slightly in Figure 7a. The effect of SLA on the PSLR and power loss is shown in Figure 7b. As mentioned above, an increase in SLA causes reduced power loss and enhanced PSLR due to increased overlapping of the SA1 and SA2 main lobes. However, when SLA is increased beyond a certain value, the grating lobes of SA1 and SA2 become so wide that they start to overlap, resulting in high side lobes and poor PSLR. Hence, SLA can be increased, to achieve an enhanced PSLR, up to a certain limit where the grating lobes start to overlap.

### 4.3. Effect of *L* for Fixed Δ and SLA

**Figure 8:**
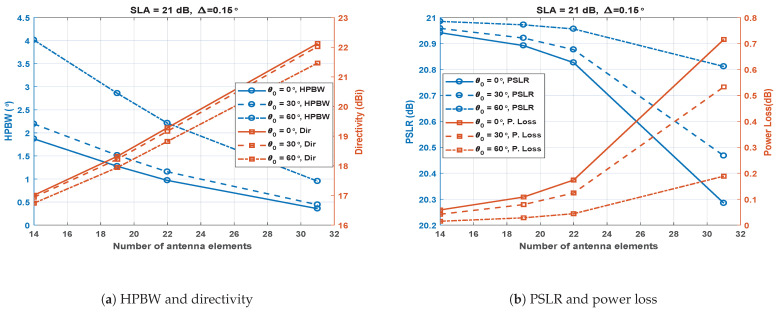
Performance metrics of the SCASS-C for variable *L*, fixed SLA = 21 dB and Δ = 0.15${}^{\circ }$.

The simulations were carried out to study the effect of the number of antenna elements (*L*) with three different cases of $\theta_{0}$ (0${}^{\circ }$, 30${}^{\circ }$ and 60${}^{\circ }$). Δ was fixed at 0.15${}^{\circ }$ and SLA was fixed at 21 dB. Figure 8a shows the results for *L* vs. HPBW. It was observed that the HPBW decreases almost linearly with an increase in *L* for all the values of $\theta_{0}$. The HPBW also increased again if the $\theta_{0}$ is increased from 0${}^{\circ }$ to 60${}^{\circ }$. Figure 8a shows the results for *L* vs. directivity. The increase in antenna elements linearly increased the directivity for all the cases of $\theta_{0}$. The directivity for 60${}^{\circ }$ is slightly lower compared to lower values of $\theta_{0}$. Figure 8b shows the effect of increasing *L* on the PSLR and power loss. With an increase in *L*, the PSLR remained almost flat for fewer antenna elements, beginning to decrease rapidly when the *L* exceed 22. A steep decaying variation was observed in the PSLR, from 20.85 to 20.3 dB with an increase in *L*. Figure 8b also shows that, with increasing *L*, the power loss increases somewhat slowly from 14 to 22 elements but increases rapidly afterwards. It is observed that the power loss is again higher at lower values of $\theta_{0}$.

The above analysis and Figure 6, Figure 7 and Figure 8 show that increasing *L* is always beneficial in terms of HPBW and directivity, similar to SULA. However, increasing *L* in SAs of SCASS-C causes narrower main lobes, causing a smaller Δ to be chosen to ensure reasonable overlapping of the main lobes. Therefore, Δ should be chosen according to the widths of the SA1 and SA2 main lobes. For wider SA main lobes, Δ can be configured in a wider range, while for narrower main lobes, Δ should be kept smaller. However, in any case, Δ should not be set to a value so large that the grating lobes overlap. It is also noteworthy that the widths of SA main lobes are affected not only by a change in *L* but also by a change in SLA. Decreasing SLA means narrower SA main lobes causing a smaller Δ to be selected and vice versa.

## 5. Conclusions and Future Work

In this paper, we presented a novel idea of staggered beam-steering of SAs for SAAs to control HPBW. For the proof of concept, the proposed technique was applied to an SCA with Dolph–Chebyshev weights. we also shed that by selecting the values of Δ and SLA appropriately, we can maintain the HPBW, PSLR, and consequently directivity of the array within reasonable limits. We also showed that the proposed array offers the narrowest main lobe among its competitors and maintains a PSLR irrespective of the steering angle. Moreover, the parametric analysis presented here identifies the appropriate range in which the parameters Δ and SLA can be adjusted to achieve a significant improvement in the performance metrics. As mentioned earlier, the appropriate values of Δ and SLA depend on the widths of the SA1 and SA2 main lobes as well as the angular separation between their grating lobes, which are further dependent on the number of elements in the SAs and their inter-element spacing. Therefore, in future, we are determined to devise a mechanism to find the appropriate values of Δ and SLA for a given set of SAs, to achieve given values of the performance metrics. It may be noted that the proposed strategy could be employed for arrays of RF and audio sensors for spatial filtering of RF or audio signals, respectively. The proposed scheme is a good choice for the situations where a pencil beam is required along with the restriction of small system size/weight, e.g., satellite-borne communication systems.

## Figures and Tables

**Table 1:** Performance metrics of the proposed and existing arrays.

Name of Array	*L*	$\theta_{0}$	Configurable Parameters	HPBW	PSLR (dB)	Dir (dBi)	Power Loss (dB)
	14	0${}^{\circ }$	SLA = 22.10 dB, $\Delta =0.2^{\circ }$	1.82${}^{\circ }$	22.0	17.24	0.1
SCASS-C	14	30${}^{\circ }$	SLA = 22.15 dB, $\Delta =0.3^{\circ }$	1.99${}^{\circ }$	22.0	17.44	0.15
	14	60${}^{\circ }$	SLA = 22.5 dB, $\Delta =0.9^{\circ }$	2.93${}^{\circ }$	22.0	17.41	0.5
	14	0${}^{\circ }$		1.89${}^{\circ }$	12.9	15.65	
SCA-U [33]	14	30${}^{\circ }$		2.18${}^{\circ }$	12.9	15.65	
	14	60${}^{\circ }$		3.88${}^{\circ }$	12.9	15.65	
	14	0${}^{\circ }$	SLA = 22 dB	2.15${}^{\circ }$	22	16.59	
SCA-C	14	30${}^{\circ }$	SLA = 22 dB	2.50${}^{\circ }$	22	16.59	
	14	60${}^{\circ }$	SLA = 22 dB	4.38${}^{\circ }$	22	16.59	
	14	0${}^{\circ }$		1.80${}^{\circ }$	13.25	17.48	
Novel CSA [30]	14	30${}^{\circ }$		2.10${}^{\circ }$	13.25	17.48	
	14	60${}^{\circ }$		3.60${}^{\circ }$	13.25	17.48	
	18	0${}^{\circ }$	SLA = 18 dB	2.94${}^{\circ }$	18	14.84	
ECSA-C [18]	18	30${}^{\circ }$	SLA = 18 dB	3.40${}^{\circ }$	18	14.84	
	18	60${}^{\circ }$	SLA = 18 dB	5.92${}^{\circ }$	18	14.84	
	16	0${}^{\circ }$		7.58${}^{\circ }$	26.4	11.6	
SULA-IWO [3]	16	30${}^{\circ }$		8.70${}^{\circ }$	26.4	11.6	
	16	60${}^{\circ }$		15.6${}^{\circ }$	15	11.6	
	32	0${}^{\circ }$		3.27${}^{\circ }$	24.6	15.12	
NULA-GO [17]	32	30${}^{\circ }$		3.77${}^{\circ }$	8.4	14.38	
	32	60${}^{\circ }$		6.57${}^{\circ }$	8.4	13.9	
	20	0${}^{\circ }$		5.30${}^{\circ }$	22.6	13	
NULA-GA [14]	20	30${}^{\circ }$		6.17${}^{\circ }$	10.2	12.44	
	20	60${}^{\circ }$		10.84${}^{\circ }$	10.2	12.1	
	21	0${}^{\circ }$		5.71${}^{\circ }$	33.3	12.84	
NULA-P [13]	21	30${}^{\circ }$		6.60${}^{\circ }$	12.62	12.7	
	21	60${}^{\circ }$		11.6${}^{\circ }$	3.8	11.25	
SULA-PSO [10]	16	0${}^{\circ }$		7.98${}^{\circ }$	27.8	11.38	
	16	30${}^{\circ }$		9.23${}^{\circ }$	27.8	11.38	
	16	60${}^{\circ }$		16.23${}^{\circ }$	13	11.38	
NULA-PSO [10]	16	0${}^{\circ }$		5${}^{\circ }$	21.08	-	

## References

[1] Balanis C.A. (2016). Antenna Theory.

[2] Langston L. (1971). Scanned directivity of linear arrays. IEEE Trans. Antennas Propag..

[3] Sun G., Liu Y., Li H., Liang S., Wang A., Li B. (2018). An antenna array sidelobe level reduction approach through invasive weed optimization. Int. J. Antennas Propag..

[4] Khalilpour J., Ranjbar J., Karami P. (2020). A novel algorithm in a linear phased array system for side lobe and grating lobe level reduction with large element spacing. Analog. Integr. Circuits Signal Process..

[5] Khan W., Saeed S., ur Rehman A. Linear Antenna-Array with Log-increasing Inter-element Spacing and Non Uniform Weights. Proceedings of the 2019 1st Global Power, Energy and Communication Conference (GPECOM).

[6] Zaman M.A., Matin M.A. (2012). Nonuniformly Spaced Linear Antenna Array Design Using Firefly Algorithm. Int. J. Microw. Sci. Technol..

[7] Khalaj-Amirhosseini M. (2019). To control the beamwidth of antenna arrays by virtually changing inter-distances. Int. J. RF Microw. Comput.-Aided Eng..

[8] Ridwan M., Abdo M., Jorswieck E. Design of non-uniform antenna arrays using genetic algorithm. Proceedings of the 13th International Conference on Advanced Communication Technology (ICACT2011).

[9] Kenane E., Djahli F. (2016). Optimum design of non-uniform symmetrical linear antenna arrays using a novel modified invasive weeds optimization. Arch. Electr. Eng..

[10] Rahman S.U., CAO Q., Ahmed M.M., Khalil H. (2017). Analysis of linear antenna array for minimum side lobe level, half power beamwidth, and nulls control using PSO. J. Microw. Optoelectron. Electromagn. Appl..

[11] Banerjee S., Mandal D. (2017). Array pattern optimization for steerable linear isotropic antenna array using novel particle swarm optimization. J. Electromagn. Waves Appl..

[12] Subhashini K.R. (2019). Antenna array synthesis using a newly evolved optimization approach: Strawberry algorithm. J. Electr. Eng..

[13] Enache F., Deparateanu D., Popescu F. Optimization of non-uniform linear antenna array with linear and parabolic parameters variations. Proceedings of the 2017 International Conference on Optimization of Electrical and Electronic Equipment (OPTIM) & 2017 Intl Aegean Conference on Electrical Machines and Power Electronics (ACEMP).

[14] Oraizi H., Fallahpour M. (2008). Nonuniformly spaced linear array design for the specified beamwidth/sidelobe level or specified directivity/sidelobe level with coupling consideration. Prog. Electromagn. Res. M.

[15] Zaman M.A., Mamun S.A., Gaffar M., Choudhury S.M., Alam M.M., Matin M.A. (2011). Phased Array Synthesis Using Modified Particle Swarm Optimization. J. Eng. Sci. Technol. Rev..

[16] Liang S., Fang Z., Sun G., Liu Y., Qu G., Zhang Y. (2020). Sidelobe Reductions of Antenna Arrays via an Improved Chicken Swarm Optimization Approach. IEEE Access.

[17] Wang H., Liu C., Wu H., Li B., Xie X. (2020). Optimal Pattern Synthesis of Linear Array and Broadband Design of Whip Antenna Using Grasshopper Optimization Algorithm. Int. J. Antennas Propag..

[18] Adhikari K., Drozdenko B. (2019). Design and Statistical Analysis of Tapered Coprime and Nested Arrays for the Min Processor. IEEE Access.

[19] Dolph C. (1946). A Current Distribution for Broadside Arrays Which Optimizes the Relationship between Beam Width and Side-Lobe Level. Proc. IRE.

[20] Senapati A., Roy J.S. (2016). Adaptive Beam Formation in Smart Antenna Using Tschebyscheff Distribution and Variants of Least MeanSquare Algorithm. Mikrotalasna Rev..

[21] Zhang Y., Wang W., Wang R., Deng Y., Jin G., Long Y. (2019). A Novel NLFM Waveform with Low Sidelobes Based on Modified Chebyshev Window. IEEE Geosci. Remote Sens. Lett..

[22] Li M., Zhang Z., Tang M.C., Yi D., Ziolkowski R.W. (2020). Compact Series-Fed Microstrip Patch Arrays Excited with Dolph–Chebyshev Distributions Realized with Slow Wave Transmission Line Feed Networks. IEEE Trans. Antennas Propag..

[23] Agha M.H., AL-Adwany M.A.S., Bayat O., Hamdoon H.T. (2023). IFT and Chebyshev-based planar array thinning for adaptive interference suppression. J. Comput. Electron..

[24] Xu S., Xu H.X., Wang Y., Xu J., Wang C., Pang Z., Luo H. (2022). Circularly Polarized Antenna Array with Decoupled Quad Vortex Beams. Nanomaterials.

[25] Xu H.X., Hu G., Han L., Jiang M., Huang Y., Li Y., Yang X., Ling X., Chen L., Zhao J. (2019). Chirality-Assisted High-Efficiency Metasurfaces with Independent Control of Phase, Amplitude, and Polarization. Adv. Opt. Mater..

[26] Xu H.X., Cai T., Zhuang Y.Q., Peng Q., Wang G.M., Liang J.G. (2017). Dual-Mode Transmissive Metasurface and Its Applications in Multibeam Transmitarray. IEEE Trans. Antennas Propag..

[27] Vaidyanathan P.P., Pal P. (2010). Sparse sensing with co-prime samplers and arrays. IEEE Trans. Signal Process..

[28] Liu Y., Buck J.R. Detecting Gaussian signals in the presence of interferers using the coprime sensor arrays with the min processor. Proceedings of the 2015 49th Asilomar conference on signals, systems and computers.

[29] Di Martino G., Iodice A. (2016). Passive beamforming with coprime arrays. IET Radar Sonar Navig..

[30] Moghadam G.S., Shirazi A.B. (2019). Novel method for digital beamforming in co-prime sensor arrays using product and min processors. IET Signal Process..

[31] Adhikari K., Buck J.R., Wage K.E. (2014). Extending coprime sensor arrays to achieve the peak side lobe height of a full uniform linear array. EURASIP J. Adv. Signal Process..

[32] Adhikari K., Buck J.R., Wage K.E. Beamforming with extended co-prime sensor arrays. Proceedings of the 2013 IEEE international conference on acoustics, speech and signal processing.

[33] Adhikari K. (2019). Beamforming with semi-coprime arrays. J. Acoust. Soc. Am..

[34] Tokan F., Gunes F. (2009). The multi-objective optimization of non-uniform linear phased arrays using the genetic algorithm. Prog. Electromagn. Res. B.

[35] Ward H. (1973). Properties of Dolph-Chebyshev Weighting Functions. IEEE Trans. Aerosp. Electron. Syst..

